# Cytotoxic furanosesquiterpenoids and steroids from *Ircinia mutans* sponges

**DOI:** 10.1080/13880209.2021.1920620

**Published:** 2021-05-27

**Authors:** Fatemeh Heidary Jamebozorgi, Morteza Yousefzadi, Omidreza Firuzi, Melika Nazemi, Somayeh Zare, Jima N. Chandran, Bernd Schneider, Ian T. Baldwin, Amir Reza Jassbi

**Affiliations:** a Department of Marine Biology, Faculty of Marine Sciences and Technology, University of Hormozgan, Bandar Abbas, Iran; b Medicinal and Natural Products Chemistry Research Center, Shiraz University of Medical Sciences, Shiraz, Iran; c Department of Biology, Faculty of Science, University of Qom, Qom, Iran; d Persian Gulf and Oman Sea Ecological Research, Iranian Fisheries Research Institute, Agricultural Research, Education and Extension Organization, Bandar Abbas, Iran; e Research Group Biosynthesis/NMR, Max Planck Institute for Chemical Ecology, Jena, Germany; f Department of Molecular Ecology, Max Planck Institute for Chemical Ecology, Jena, Germany

**Keywords:** Furanosesquiterpenes, marine sponge, cholesterol, ergosterol, stigmasterol, γ-sitosterol

## Abstract

**Context:**

*Ircinia mutans* Wilson (Irciniidae) is a sponge with antimicrobial and cytotoxic constituents.

**Objective:**

Our objective was to characterise the cytotoxic constituents of two seasonal collections of *I. mutans*.

**Materials and methods:**

The sponges were extracted in methanol-dichloromethane and their constituents were purified and characterised using column chromatography, GC-MS, 1 D and 2 D NMR. Anti-proliferative activities of the compounds, were evaluated using 3-(4,5-dimethylthiazol-2-yl)-2,5-diphenyltetrazolium bromide (MTT) colorimetric assay (0.25–100 μg/mL, 72 h) against leukaemia (MOLT-4), breast (MCF-7) and colon cancer (HT-29) human cells.

**Results:**

Three furanosesquiterpoids; furodysin (**1**), *ent*-furodysinin (**2**) and furoircin (**3**) and ten sterols were characterised in *I. mutans*, for the first time. Cholesterol (**4**), cholesta-5, 7-dien-3β-ol (**5**) and ergosterol (**6**) were determined in the sponge from the winter collections, while cholesta-5, 22-dien-3β-ol (**7**), 24-methyldesmosterol (**8**), campesterol (**9**), stigmasterol (**10**), γ-ergostenol (**11**), chondrillasterol (**12**) and γ-sitosterol (**13**) were detected in the summer samples. The steroids from the winter collection exhibited cytotoxic activity with IC_50_ values of 13.0 ± 0.9, 11.1 ± 1.7 and 1.1 ± 0.4 µg/mL, against the mentioned cancer cell lines, respectively, while those from the summer sample, showed greater activity, IC_50_ = 1.1 ± 0.2 μg/mL against MOLT-4. The purified steroids showed potent MOLT-4 cytotoxic activity, IC_50_ values = 2.3–7.8 µg/mL.

**Discussion and conclusion:**

The present study suggests that *I. mutans* is a rich source of cytotoxic steroids, and introduces **3** as new natural product. Considering the high cytotoxic activity of the steroids, these structures could be candidates for anticancer drug development in future research.

## Introduction

Sponges (Phylum Porifera) are the most primitive multicellular eukaryotic organisms. They are sessile without defense organs and therefore produce different secondary metabolites to protect themselves from predators and pathogens in their environment (Proksch 1994). The genus *Ircinia* Nardo (Irciniidae) comprises about 73 species which have been widely investigated for drug discovery. This genus of sponge is a rich source of a variety of steroids, terpenoids and other types of secondary metabolites with different biological activities (Faulkner 1973; Emura et al. 2006; Xu et al. 2008; Hahn et al. 2014; Yang et al. 2014).

Steroids are the important class of metabolites that have two main biological functions: structural roles, such as with cholesterol which is an important component of cell membrane, and physiological roles such as signalling molecules that activate steroid hormone receptors (Fahy et al. 2005). Most marine organisms have been found to be sources of unusual steroid metabolites, but some researchers believe that marine sponges may provide the most diverse steroids in the entire animal kingdom (Aiello et al. 1999). The steroids isolated from marine sponges show promising cytotoxic activity against different cancer cell lines. Aiello and colleagues (1999) reported in their review that steroids from sponges are potent anticancer, antiviral, antibacterial, and antifungal agents. Cytotoxic bioassay-guided purification of steroid-rich fractions of non-polar solvent extracts of *Axinella sinoxea* Alvarez & Hooper (Axinellidae), resulted in the identification of eight steroids including: cholesta-5,22-dien-3β-ol, cholest-5-en-3β-ol (cholesterol; **4**), ergosta-5,22-dien-3β-ol, ergost-5-en-3β-ol (**9**), stigmasta-5,22-dien-3β-ol (stigmasterol; **10**), γ-sitosterol (clionasterol; **13**), 33-norgorgosta-5,24(28)-dien-3β-ol and stigmasta-5,24(28)-dien-3β*-*ol. The cytotoxic activity of the compounds was measured using the 3-(4,5-dimethylthiazol-2-yl)-2,5-diphenyltetrazolium bromide (MTT) colorimetric assay against three human cancer cell lines MOLT-4, MCF-7 and HT-29, and showed that the steroids are potent cytotoxic agents with IC_50_< 10 µg/mL (Jamebozorgi et al. 2019). A series of new 5,6-epoxysterols isolated from a Chinese species, *Ircinia aruensis* Hentschel have shown moderate to strong cytotoxic activity on H-460, LOVO and MCF-7 cell lines (Xu et al. 2008). Also, Trinh et al. (2018), isolated six new 9α-hydroxy-5α,6α-epoxysterols from the Vietnamese marine sponge, *Ircinia echinate* and showed that two sterols (24 *R*)-5α,6α-epoxy-24-ethyl-cholesta-7-en-3β,9α-diol and 5α,6α-epoxycholesta-7-en-3β,9α-diol have cytotoxic activity against three human cancer cell lines, MCF-7, Hep-G2 and LU-1.

Furanosesquiterpenes are an outstanding group of natural products that have been isolated from both terrestrial and marine organisms including sponges, cnidaria (especially class anthozoa), and molluscs (Fontana et al. 1995; Arepalli et al. 2009; Rajaram et al. 2013). Tubipofuran and 15-acetoxytubipofuran are examples of cytotoxic marine furanosesquiterpenoids, isolated from the organ pipe coral (*Tubipora musica* Linnaeus, Tubiporidae), which have shown ichthyotoxicity against killifish *Oryzias latipes* (IC_50_ 15 μg/mL and 20 μg/mL respectively), and the latter has also shown cytotoxicity against B-16 melanoma cells *in vitro* (IC_50_ 33 μg/mL) (Iguchi et al. 1986). (-)-Furodysinin is antiparasitic against the tested organism *Nippostrongylus brasiliensis* Travassos (Heligmonellidae) *in vitro* but inactive against two others *N. dubius* Baylis (Heligmosomidae) and *Hypselodoris nana* (Chromodorididae) *in vivo* test above 1000 ppm (Horton et al. 1990). The furanoid-sesquiterpenes furodysin and furodysinin previously were isolated from sponges *Dysidea herbacea* Keller (Dysideidae) and *Dysidea fragilis* Montagu (Dysideidae) (Kazlauskas et al. 1978; Dunlop et al. 1982; Su et al. 2010), but to the best of our knowledge, their cytotoxic activity has not been investigated.

Several researchers have investigated the effects of different ecological and biological factors on the production of secondary metabolite and announced that variations in the natural products production patterns can be explained by the effect of these biotic and abiotic factors. Sacristán-Soriano et al. (2012), by examining the temporal trends in the secondary metabolite production of the sponge *Aplysina aerophoba* Nardo (Aplysinidae), showed that concentrations of bromo-alkaloid compounds in ectosome layer during the summer are more than those in the winter season and that the levels of secondary compounds from the outer layer of the sponge, were significantly correlated with water temperature. Abdo et al. (2007), studied the effect of temperature and spatiotemporal variation on production of salicylihalamide A in the sponge *Haliclona* sp. Grant (Chalinidae) and found environmental and physiological factors to be important in the production of this compound. They showed that “salicylihalamide A” concentration was significantly correlated with water temperature and was significantly higher in summer than in the winter collections in sponges from Bremer Bay. Turon et al. (1996) suggested that the greater variety of natural compounds in the warmer seasons of the year may have been a response to increased activity of competitors, while Duckworth and Battershill (2001), implicated fouling from other organisms on the sponge surface.

Previously, different solvent extracts of *I. mutans* were subjected to different bioassays such as antibacterial (Nazemi et al. 2014), antifungal (Nazemi et al. 2013), and cytotoxic against human cancerous cell lines (Jamebozorgi et al. 2018; Nazemi et al. 2020), but to the best of our knowledge there is no report on chemical characterisation and biological evaluation of the isolated compounds from this species in the literature. We report here, for the first time, the structure elucidation of cytotoxic steroids and furanosesquiterpenoids from *I. mutans* collected from the Persian Gulf during two seasons.

## Materials and methods

### Instrumentation and reagents

NMR spectra of the purified compounds were recorded on a Bruker Avance 500 spectrometer (Bruker Biospin, Karlsruhe, Germany), operating at resonance frequencies of 500 MHz for ^1^H and 125 MHz for ^13^C, respectively. Standard Bruker pulse sequences were used for measuring ^1^H NMR, ^13^C APT, ^1^H-^1^H COSY, ^1^H-^13^C HSQC and ^1^H-^13^C HMBC spectra. Tetramethylsilane (TMS) was used as an internal standard for referencing ^1^H and ^13^C NMR spectra. Data acquisition and processing was accomplished using Bruker Topspin 2.1. EI-MS spectra were recorded on an Agilent 5975 C inert GC/MS instrument. For isolation and purification of the sponge’s metabolites, different chromatographic separations were performed, including silica gel open column chromatography (CC: 0.063–0.200 mm particle size), flash column chromatography (FCC: 0.040–0.063 mm particle size) and TLC using silica gel 60 F_254_ pre-coated plates (0.25 mm film thickness). The adsorbents were purchased from Merck, Darmstadt, Germany. For further purification of the fractions, reversed-phase (RP-18) HPLC analyses were performed using a Knauer semi-preparative HPLC with a K-1050 pump and a four wavelength K-2600 UV detector set at λ 210 nm (Jassbi et al. 2014a). The semi-preparative HPLC column (Phenomenex RP-18, 250 × 10 mm) was eluted with 95% acetonitrile (solvent B) and 5% in ultrapure water (solvent A). The flow rate of the mobile phase was set at 4.5 mL/min.

### Collection of the sponges

*Ircinia mutans* was collected by scuba diving in January 2015, and June 2016 at a depth of 10–13 m near Larak Island in the Persian Gulf. Samples were placed immediately in plastic bags containing seawater and transferred to the laboratory on ice and then stored at −20 °C after other organisms, such as brittle sea star, bivalves, barnacles, oligo- and poly-chaetes, were removed. One of us, M. Nazemi, the marine biologist, identified the sponge sample, based on scanning and optical microscope studies of skeletal slides and dissociated spicule mounts with the sponge taxonomic keys developed by John N.A. Hooper (2000). Parts of the sponge were stored in 70% ethanol and kept in Medicinal and Natural Products Chemistry Research Center, Shiraz University of Medical Science museum, as a voucher specimen; 93–7–1-1/1.

### Extraction of the sponge

The sponge sample collected in winter (W) (652 g fresh weight) was cut into small pieces (approximately 1 cm) and extracted by methanol (2 × 4 L) for 4 days followed by the same volumes of dichloromethane (DCM) continuously at room temperature in the dark to afford residues of MeOH (3.2 g) and DCM (1.4 g) extracts after evaporation of the solvents in reduced pressure at 40 °C. The fresh sponge sample (3.29 kg) collected in summer (S) was extracted by MeOH and DCM as described earlier (Jamebozorgi et al. 2019). The solvent of the DCM (8.5 g) extract was evaporated under reduce pressure at 40 °C. The methanol extract (500 mL) of the summer sample was subjected to liquid-liquid extraction (LLE), using *n*-hexane (3 × 250 mL), DCM (3 × 250 mL), and 1-butanol (3 × 200 mL) to afford three fractions: *n*-hexane (5.05 g), DCM (1.92 g), and 1-butanol after evaporation of the solvents in vacuum. On the basis of TLC examination, we choose the hexane fraction for further phytochemical investigation.

### Isolation and purification of the chemical constituents

#### Isolation of the winter collected sponges’ extract

The MeOH and DCM extracts of the winter samples were mixed based on their similar patterns from the TLC analyses. The combined extracts were subjected to silica gel-FCC (50 × 4 cm; 100 g). The column was eluted with *n*-hexane with stepwise increasing the polarity of the mobile phase to ethyl acetate (EtOAc) and then to pure methanol to afford 24 fractions. Compound **1** (240 mg) was obtained as a colourless oil and detected as a UV quenching spot (*R_f_* = 0.5) on silica gel F_254_ TLC using 100% *n*-hexane.

The fractions eluted by *n*-hexane: EtOAc (9: 1); FW9 and 10 (118 mg) were pooled based on their compositional similarity. After TLC analyses, they were subjected to FCC (40 × 2 cm) over silica gel (23 g, 230–400 mesh) using different portions of hexane, DCM and methanol for further purification. The resulting fractions F51-80 (compound **4–6**; 49 mg) were pooled and dried under reduced pressure. The silica gel TLC using DCM: MeOH (98: 2, *R_f_* = 0.5) of FW9-10-F51-80 exhibited a single UV-quenching spot. The isolated compound was subjected to ^1^H and ^13^C NMR spectroscopy and GC-MS analyses and found to contain at least three major steroids **4–6**.

#### Isolation of the summer collected sponges’ extract

**Figure 1.**
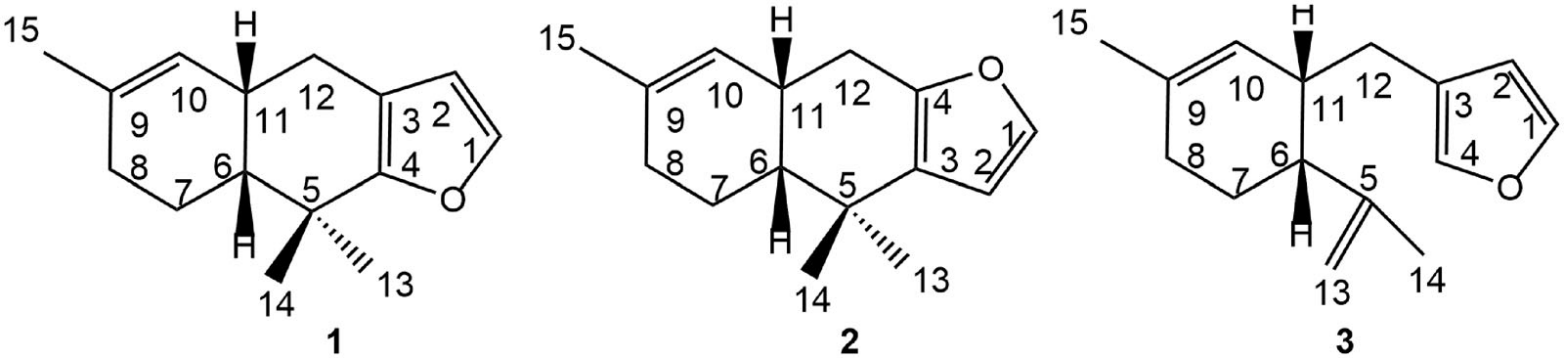
Structures of furodysin (**1**), furodysinin (**2**), and furoircin (**3**) isolated from *Ircinia mutans*.

The hexane layer of the methanol extract of the summer-sponge sample was subjected to FCC (50 × 4.5 cm) over AgNO_3_-impregnated (7.5 g) silica gel (150 g). The column was eluted with different portions of *n*-hexane and DCM followed by MeOH to afford 32 fractions. Compound **1–3** were detected on TLC in the hexane rich fractions as described above and were further purified with RP18 semi preparative HPLC with the retention times of 7.48, 7.75 and 8.42 min, respectively (Figure 1).

**Figure 2.**
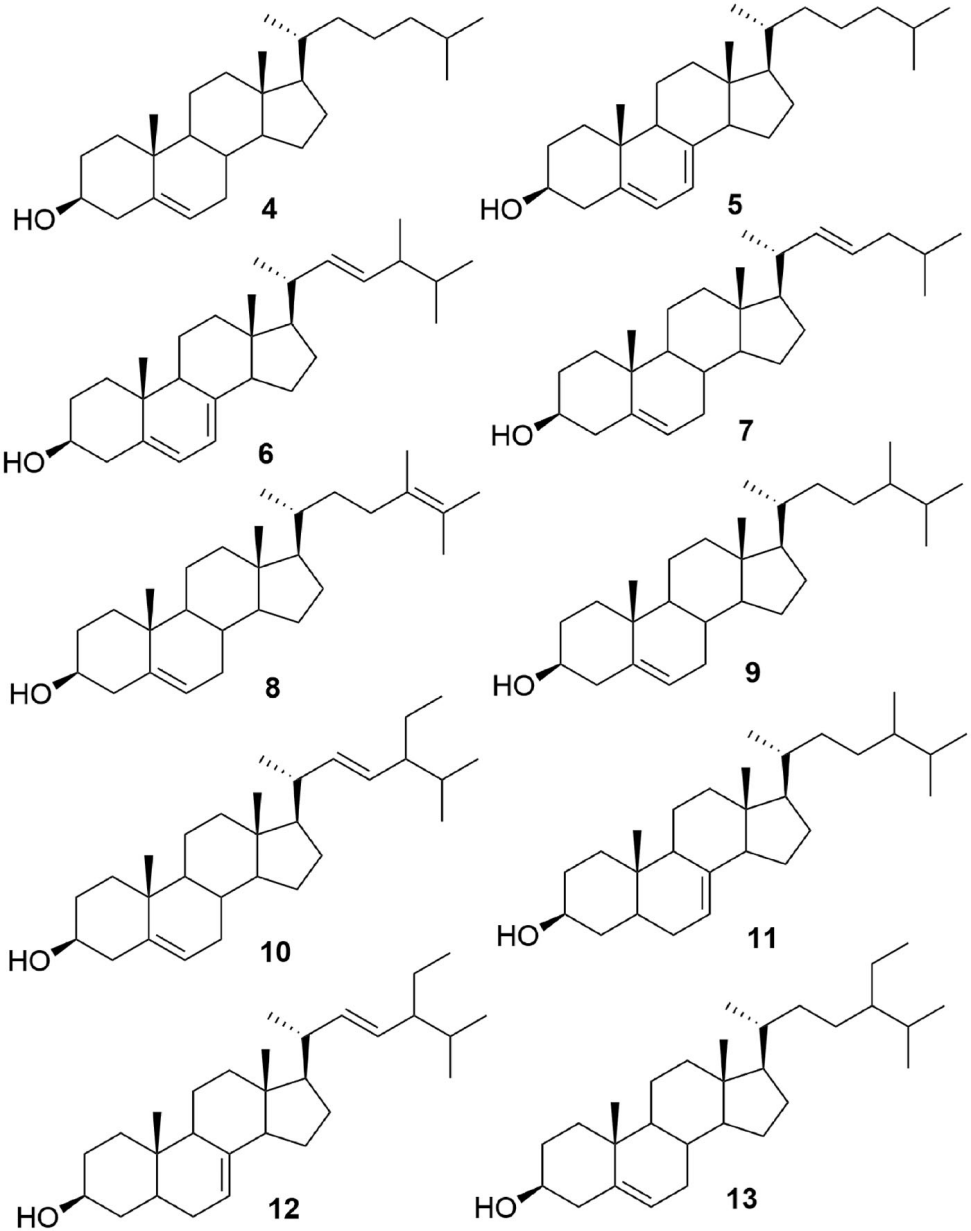
Structures of steroids identified from *Ircinia mutans*.

**Figure 3.**
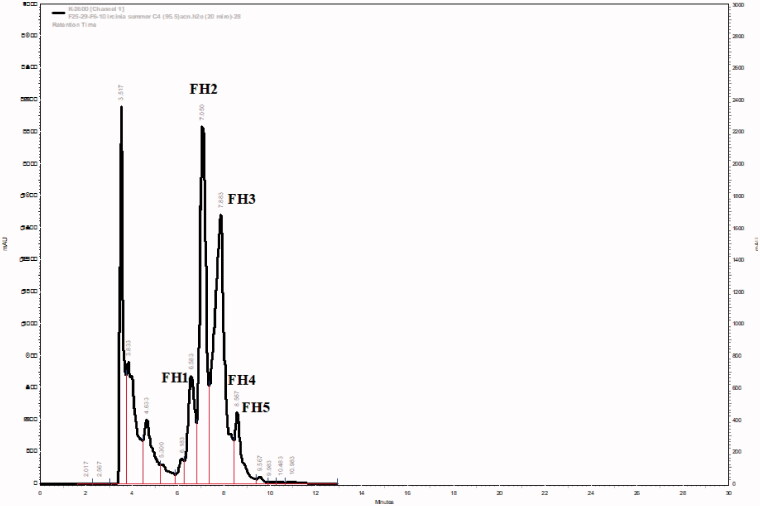
HPLC chromatograms of the subfraction FS6-10- F25-29 (steroids fraction) isolated from *I. mutans* (summer sample).

**Figure 4.**
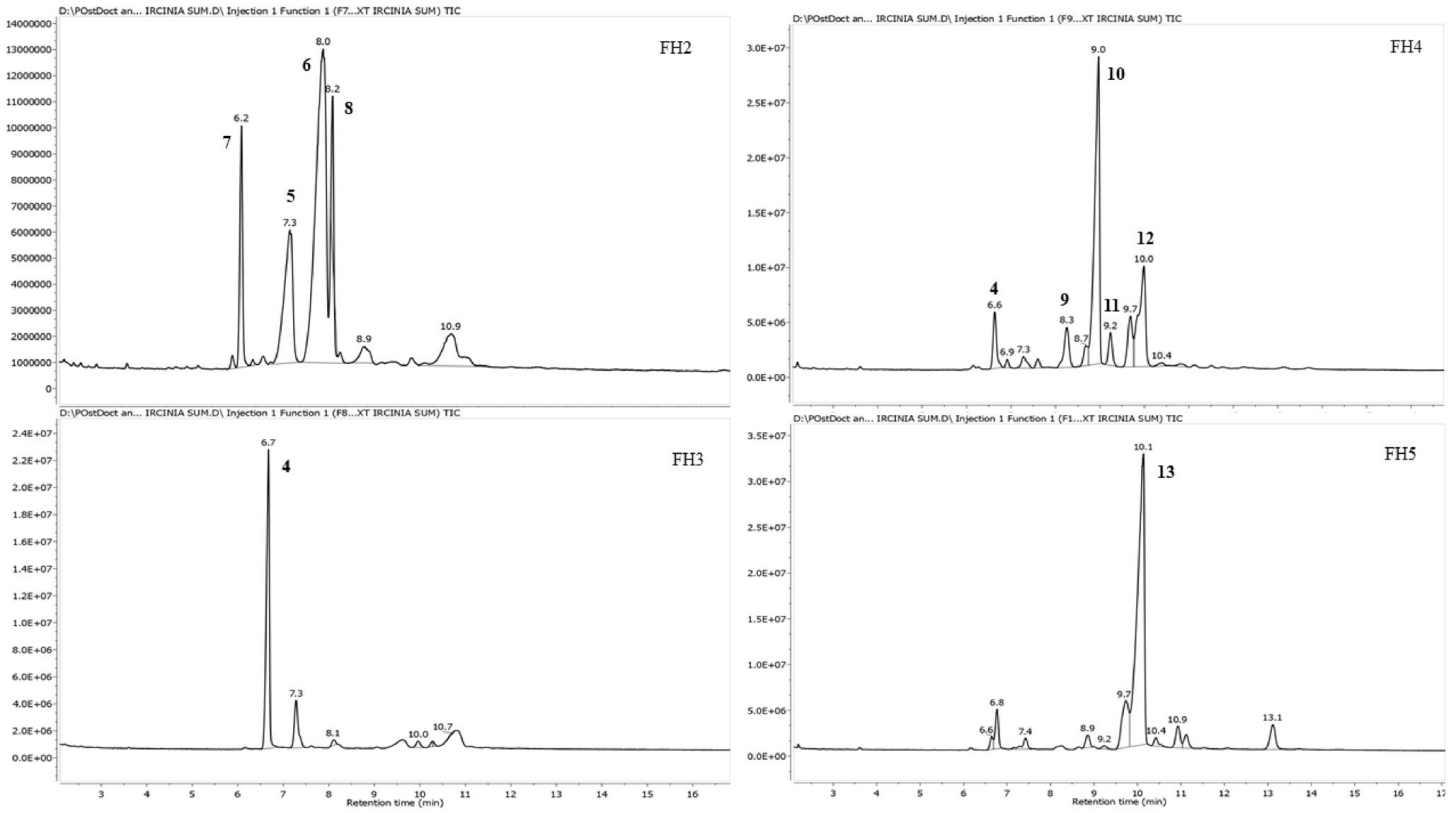
GC-MS chromatograms of FH2-FH5 of the HPLC semi-purified compounds **4–13** from FS6-10- F25-29 (steroids fraction) isolated from *I. mutans* (summer sample).

Repeated silica gel (45 g)-FCC (50 × 3 cm) of fractions F6-F10 (300 mg) as described for that of the winter sample yielded a pale-yellow powder (FS6-10- F25-29) after removal of the eluting solvents (98 mg). The fraction was further analysed by GC-MS for characterisation of its constituents; **4–13** (Figure 2). For further purification of this fraction, reversed-phase (RP-4) HPLC analyses were performed using the same HPLC system described earlier. The HPLC column (Eurospher-100-5 C4, 250 × 4.6 mm, Knauer, Germany) was eluted with 95% acetonitrile and 5% in ultrapure water. The flow rate of the mobile phase was set at 1 mL/min. The major peaks were collected as FH2 (3.5 mg) FH3 (7.6 mg), FH4 (0.6 mg) and FH5 (0.8 mg) (Figure 3). These fractions appeared to be pure based on analytical HPLC chromatograms and were subjected to GC-MS for their identification and qualitative analyses (Figure 4) and further cytotoxic tests.

#### Gas chromatography–mass spectrometry (GC-MS)

GC-MS analyses were performed using an Agilent 7890 A GC coupled to HP-6890 mass spectrometer operating in EI mode at 70 eV. The GC was equipped with a DB-5 MS and HP-5 MS (J & W Scientific column, 30 m × 0.25 mm i.d., 0.25 μm film thickness) for the winter and the summer samples, respectively. To analyse the steroids, the oven temperature was set at 265 °C for 20 min, increased to 300 °C at 5 °C/min and kept for 10 min at the final temperature. Helium (He) was used as the carrier gas with a flow rate of 1 mL/min. The injector temperature was set at 300 °C, in the split mode (1:10). The injection volume was 0.1 μL for all of the samples (Jassbi et al. 2013).

#### Derivitization of the steroids to trimethylsilyl derivatives

In order to quantify the steroid contents in the sub-fractions FW9-10- F51-80 of the winter sample by GC-MS, fraction constituents were transformed to trimethylsilyl derivatives. Briefly, 200 μL of the reagent, *N*-*O*-bis-(trimethylsilyl)-trifluoroacetamide (BSTFA) containing 1% trimethylsilyl chloride (TMCS) was added to 2 mg of F9,10 -F51-80 and vortexed well. Afterwards, the mixture was heated in a hot water bath (60 °C) for 1 h. Samples were evaporated under a stream of nitrogen gas, redissolved in 200 μL DCM and the resulting solution was subjected to GC-MS as previously described (Jassbi et al. 2013).

#### Cytotoxicity assays

MOLT-4 (human lymphoblastic leukaemia, Cell bank number: C149) and MCF7 (human breast adenocarcinoma, Cell bank number: C135) cells were obtained from National Cell Bank of Iran, Pasteur Institute, Tehran, Iran. HT-29 (human colorectal adenocarcinoma, Cell bank number: IBRC C10097) cell line was purchased from Iranian Biological Resource Centre, Tehran, Iran. MOLT-4 and MCF-7 cells were cultured in RPMI 1640 medium supplemented with 10% v/v foetal bovine serum, penicillin (100 units/mL) and streptomycin (100 μg/mL), and HT-29 cells were cultured in DMEM medium supplemented with 10% v/v foetal bovine serum, penicillin (100 units/mL) and streptomycin (100 μg/mL) and l-glutamine at 37 °C in humidified air containing 5% CO_2_.

*In vitro* cytotoxic activity of compounds was evaluated by using the MTT reduction assay. MTT is a rapid colorimetric assay that can be used to measure cytotoxicity, proliferation or activation of the cells (Mosmann 1983). The purified compounds were dissolved in DMSO and isopropanol, and then diluted in growth medium at least 400 times. Cells were seeded in 96-well plates at the density of 50,000 cells/mL (MOLT-4 cell line), 25,000 cells/mL (MCF-7 cell line) and 10,000 cells/mL (HT-29 cell line) in 100 μL medium and incubated for 24 h at 37 °C. Then, four different concentrations of each fraction and cisplatin (as positive controls) were added to the wells of the plates in triplicate and they were incubated at 37 °C for 72 h. Afterwards, the media (80 µL) of each well was replaced by 80 µL MTT solution diluted in RPMI without phenol red (concentration of 0.5 mg/mL) and incubated at 37 °C. After 4 h incubation and formation of formazan crystals, the media was removed and 200 μL DMSO was added to each well to dissolve the crystals. Finally, absorbance was measured at a wavelength of 570 nm with background correction at 650 nm using a microplate reader (model 680, Bio-Rad, Japan) and IC_50_ (concentration that results in 50% inhibition of cell viability) for each compound was calculated with Curve Expert statistical analyses (Jassbi et al. 2014b).

#### Spectroscopic data of the compounds

*Furoircin* (**3**): colourless perfumed oil (3.8 mg.). [α]_D_^19^ +240 (c 0.0015 g/mL, CHCl_3_), EIMS (resulting from GC-MS) *m/z* (rel. int.%): 216 [M]^+^ C_15_H_20_O (26), 201 [216 – CH3]^+^ (6), 147 (7), 135 (41), 133 (16), 119 (21), 115 (11), 107 (100), 105 (29), 93 (84), 91 (61), 81 (30), 79 (35), 77 (44), 67 (17), 55 (22), 53 (35), 41 (23). For ^1^H NMR (500 MHz, CDCl_3_) and ^13^C NMR (125 MHz, CDCl_3_) spectroscopic data see in Table 1.

**Table 1.** ^1^H NMR (500 MHz) and ^13^C NMR (125 MHz) data of furoircin (**3**) in CDCl_3_.

position	δ_H_ (*J* in Hz)	^1^H-^1^H COSY	δ_C_ (HSQC)	HMBC
1	7.34 brt (*J* = 1.8)	H-2	142.46	C-2, C-3, C-4
2	6.24 brd (*J* = 1.7)	–	111.49	C-1, C-3, C-4, C-12
3	–	–	123.82	–
4	7.17 brs	H-2, H-11	139.40	C-1, C-2, C-3, C-11, C-12
5	–	–	147.63	–
6	2.24 ddd (*J* = 11.9, 3.8, 3.8)	H-7	43.80	C-5, C-7, C-8, C-11, C-12, C-13
7	1.68 m	–	22.50	C-5, C-6, C-8, C-9, C-11
8	2.00 m	H-7	30.78	C-6, C-7, C-9, C-10
9	–	–	133.85	–
10	5.39 brdd (*J* = 1.8, 1.8)	H-11, H-12′	124.94	C-6, C-8, C-11, C-15
11	2.41 m	H-12′	36.77	C-2, C-4, C-9, C-10, C-12, C-15
12	2.38 dd (*J* = 12.6, 3.9)	–	25.97	C-2, C-3, C-4, C-6, C-11
12′	2.07 ddd (*J* = 12.7, 12.7, 2.6)	–	25.97	C-2, C-3, C-4, C-6, C-11
13	4.87 brs	H-14	110.14	C-5, C-6, C-11(w*), C-14
13′	4.72 brs	H-14, H-6	110.14	C-5, C-6, C-11(w), C-14
14	1.77 s	–	22.81	C-5, C-6, C-13
15	1.63 s	–	23.55	C-8, C-9, C-10, C-11, C-14

*Weak cross peak.

The spectroscopic data of previously described compounds are presented in the supplemental material.

#### Statistical analysis

The normality of data and the homogeneity of variances were surveyed with Kolmogorov–Smirnov and Leven’s tests, respectively, and log10 transformation of some data was necessary to achieve analysis requirement. Then, the effect of each fraction on cell line were compared by means of one-way ANOVAs, and the mean comparison was conducted with the Duncan’s test at 5% significant level for steroid’s sub fractions. To compare the effect of steroids extracted from two seasons on MOLT-4 cell line, the independent sample *t*-test was used between the mean activity of winter and summer samples, where the assumption of equal variances was met. Significance levels in all tests was 5% (*p* < 0.05) and data were expressed as mean ± SD. Data were analysed using SPSS statistical software (USA, release 16).

## Results

The DCM-MeOH extracts of the winter and summer sponge samples were subjected to repeated FCC on silica gel and HPLC to yield compounds **1–3** (Figure 1) and **4–13 (**Figure 2). The chemical structures of the compounds were elucidated using different spectroscopic data including 1 D and 2 D NMR, EI-MS, UV and GC-MS and comparing these data with those reported in the literature. Compound **3** was previously synthesised as a precursor of **1** (Vaillancourt et al. 1991), but it is a new natural product and its NMR spectral signals were not fully assigned.

### Structure elucidation of compound **3**

In the ^1^H NMR, three signals at δ 7.34 (brt, *J* = 1.8 Hz), 6.24 (brd, *J* = 1.7 Hz), and δ 7.17 (s) suggested a mono-substituted furan ring in the molecule. An isopropenyl group represented by two broad singlet signals at δ 4.87 and 4.72 and an olefin methyl at δ 1.77. The signals of a tri-substituted cyclohexene moiety were similar to those reported for furodysin represented by an olefin methyl group signal at δ 1.63 which is coupled with an allylic methine at δ 5.39 (br.dd, *J* = 1.8, 3.8 Hz), together with two methylene at δ 1.68 and 2.0 and two methine signals at δ 2.41 and 2.24. A methylene signal at δ 2.38 (dd, *J* = 12.6, 3.9 Hz) and 2.07 (ddd, *J* = 12.7, 12.7, 2.6 Hz) attached the cyclohexene ring to the furan ring. Using the APT ^13^C NMR and HSQC spectra, 15 signals representing four CH_2_, six CH, two CH_3_ and three quaternary carbon atoms suggested the structure of a sesquiterpene for compound **1**. The connectivity of the proton signals to their respective attached carbon atoms were confirmed by an HSQC experiment. Three methine and one quaternary carbon signals at δ 142.5, 111.5, 139.4 and 123.8, respectively, represented the mono-substituted furan ring, the signals of the isopropenyl resonated at δ 22.8 (CH_3_), 110.1 (CH_2_) and 147.6 (C). In the HMBC spectrum the cross peaks between H-12 and C-3 and C-11 confirmed the connectivity of the furan and cyclohexene rings via C-12, and the cross peaks between H-6 and C-5 and H-15 and C-9 and C-10 determine the position of the methyl and isopropenyl group on the cyclohexene ring. Two small (*J* = 3.8 Hz) and one big (*J* = 11.9 Hz) coupling constants for H-6 at δ 2.24 and a weak cross peak between H-14 and H-12 in the NOESY spectrum was compatible with the *cis* configuration at C-6 and C-11 substitution (Table 1).

### Steroids from winter sample (compounds **4–6**)

GC-MS and ^13^C NMR analyses of sub fraction FW9-10- F51-80 of winter sample revealed that this sponge is a rich source of C_27_ and C_28_ sterols. The sub-fraction, despite its TLC analysis suggesting its purity, consisted of three major Δ^5^-steroids including; cholest-5-en-3β-ol (cholesterol) (**4**), cholesta-5, 7-dien-3β-ol (**5**) and ergosta-5, 7, 22-trien-3β-ol (ergosterol) (**6**) (NIST Chemistry Web Book, Pub Chem.ncbi and The Pherobase Tools). The structures of the steroids were elucidated by GC-MS and ^13^C NMR spectral data analyses, which were confirmed by comparison to those previously reported in the literature for the authentic compounds. The presence of Δ^5^-en, Δ^5,7^-dien and Δ^5,7,22^-trien were confirmed by the ^13^C-NMR spectra which exhibited signals in the sp^2^ carbon region (δ 116.25–141.40). The chemical shifts of the ^13^C NMR signals of the sub-fraction FW9-10- F51-80 (**4–6**) are consistent with the spectroscopic data previously reported for the above mentioned compounds (Batta et al. 1995; Plouguerné et al. 2006; Zhao et al. 2012).

### Steroids from summer sample (compounds 4-13)

Five subfractions (FH1-FH5) which were collected from HPLC column of the steroidal fraction (FS6-10- F25-29), and their analysis by GC-MS showed that the variety of steroids was greater than that of the winter sample. The above sub-fractions consisted of about ten Δ^5^, Δ^7^, and Δ^5,7^ steroids, including, **4**, **5**, **6**, cholesta-5, 22-dien-3β-ol (**7**), ergosta-5, 24-dien-3β-ol (24-methyldesmosterol) (**8**), ergost-5-en-3β-ol (campesterol) (**9**), stigmasta-5,22-dien-3β-ol (stigmasterol) (**10**), *γ*-ergostenol (fungisterol) (**11**), 5*α*-stigmasta-7,22-dien-3β-ol (chondrillasterol) (**12**), and *γ*-sitosterol (clionasterol) (13). GC-MS identification of the sterols was done by comparing their GC relative retention index calculated on a non-polar capillary column relative to a series of normal alkane (C_21_-C_40_) and their mass spectra compared with those reported in data banks (NIST Chemistry Web Book, Pub Chem.ncbi and The Pherobase Tools) (Table 2). The results of GC-MS analyses of the fractions showed that FH2 constitutes compounds **7** (10.2%), **5** (13.1%), **6** (49.3%), **8** (12.9%); FH3 contained compound **4** (60.8%); FH4 constituted of compounds **4** (6.3%), **9** (6.3%), **10** (45.7%), **11** (4.4%), **12** (15.9%) and finally FH5 presented compounds **13** (65.8%) as its major constituent (Table 3).

**Table 2.** Sterol composition of *I. mutans* detected by GC-MS.

Compound	Retention time (min)	Kovats retention index (KI) Column type (HP-5 MS)	Kovats retention index standard (Colum stationary type)	Base peak (*m/z*)	Molecular weight	Reference for identification
cholesta-5, 22-dien-3β-ol (**7**)	6.18	3087	3070 (active phase: methyl Silicone*)	55	384	webbook.nist.gov
cholest-5-en-3β-ol (cholesterol) (**4**)	6.67	3121	3192 (active phase: DB-5*)	43	386	Pherobase.com
cholesta-5, 7-dien-3β-ol (**5)**	7.27	3158	3160 (active phase: methyl Silicone *)	351	384	webbook.nist.gov
ergosta-5, 7, 22-trien-3β-ol (ergosterol) (**6**)	8.01	3204	3152 (active phase: DB-5*)	363	396	webbook.nist.gov
ergosta-5, 24-dien-3β-ol (24-methyl-desmosterol) (**8**)	8.22	3214	3230 (active phase: OV-1*)	314	398	webbook.nist.gov
ergost-5-en-3β-ol (campesterol) (**9**)	8.26	3216	3131 (active phase: HP-5 MS*)	43	400	webbook.nist.gov
stigmasta-5,22-dien-3β-ol (stigmasterol) (**10**)	8.97	3252	3248 (active phase: VF-5MS*) 3170 (active phase: HP-5 MS*)	412	412	webbook.nist.gov
γ-ergostenol (fungisterol) (**11**)	9.24	3265	3220 (active phase: Methyl Silicone*)	400	400	webbook.nist.gov
5α-stigmasta-7,22-dien-3β-ol (chondrillasterol) (**12**)	9.69	3288	3295 (active phase: DB-5MS*)	271	412	webbook.nist.gov
γ-sitosterol (clionasterol) (**13**)	10.13	3308	3351 (active phase: HP-5 MS*)	43	414	webbook.nist.gov

*Column type: VF-5MS; DB-5; HP-5 MS: 5% phenylmethyl polysiloxane, OV-1, DB1 = 100% methylsiloxane

**Table 3.** Sterol composition of the HPLC Purified fractions from FS6-10- F25-29, characterised by GC-MS.

Fraction	Compounds
FH2	Cholesta-5, 22-dien-3β-ol (**7**) (10.2%)*
	Cholesta-5, 7-dien-3β-ol (**5**) (13.1%)*
	Ergosta-5, 7, 22-trien-3β-ol (ergosterol) (**6**) (49.3%)*
	Ergosta-5, 24-dien-3β-ol (24-methyldesmosterol) (**8**) (12.9%)*
FH3	Cholest-5-en-3β-ol (cholesterol) (**4**) (60.8%)*
FH4	Cholest-5-en-3β-ol (cholesterol) (**4**) (6.3%)*
	Ergost-5-en-3β-ol (campesterol) (**9**) (6.3%)*
	Stigmasta-5,22-dien-3β-ol (stigmasterol) (**10**) (45.7%)*
	γ-Ergostenol (fungisterol) (**11**) (4.4%)*
	5α-Stigmasta-7,22-dien-3β-ol (chondrillasterol) (**12**) (15.9%)*
FH5	γ-Sitosterol (Clionasterol) (**13**) (65.8 %)*

*Area percentage (%) of the detected compounds using GC-MS.

### Cytotoxicity

**Figure 5.**
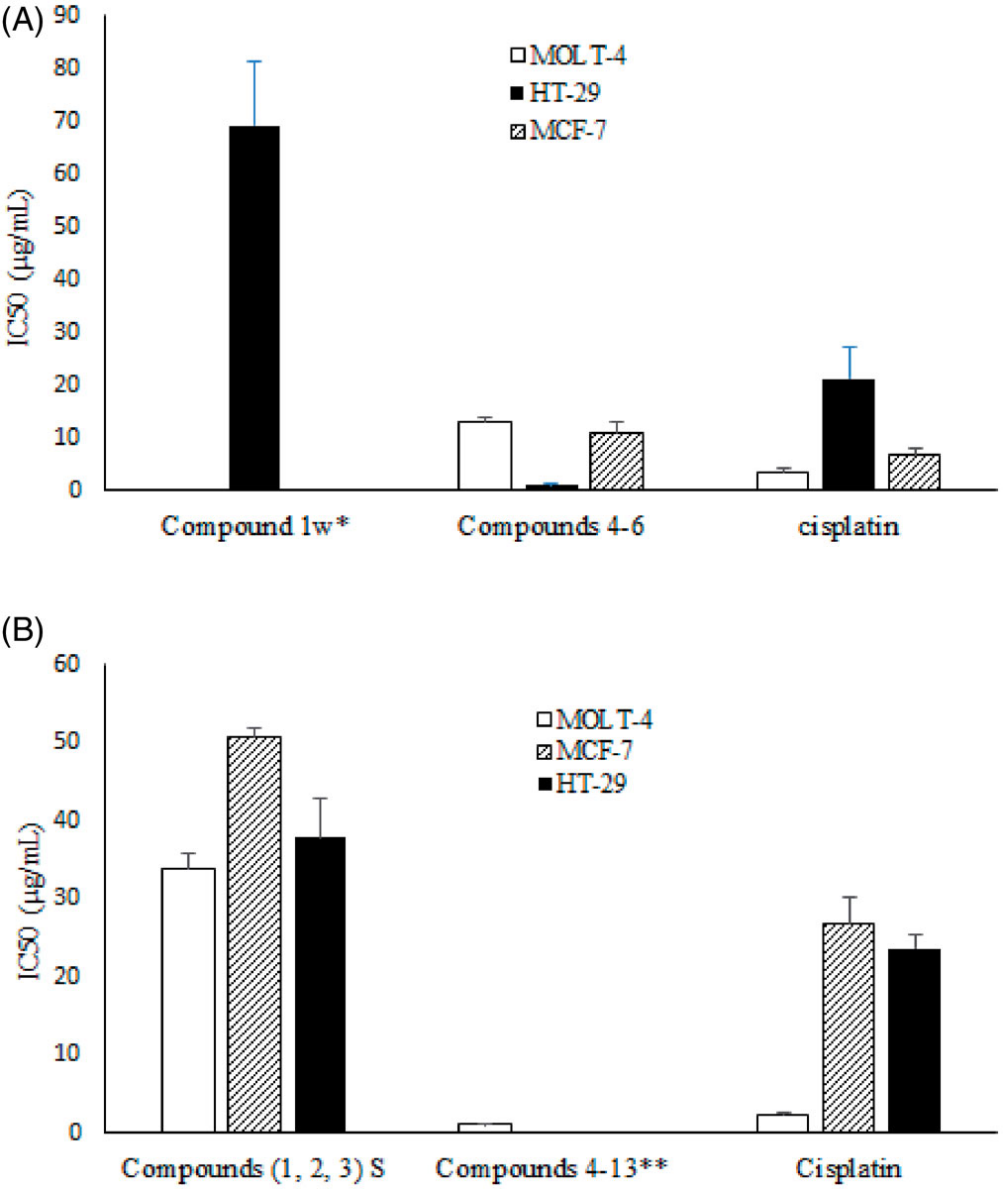
Cytotoxic activity of compounds isolated from *I. mutans* collected in winter (A) and summer (B) against three human cancer cell lines. Values are presented as mean ± S.E.M. of 3–5 experiments. *Compound 1w was not active against MOLT-4 and MCF-7 cell lines; IC_50_ > 100 µg/mL. **Compounds **4–13** were not tested against MCF-7 and HT-29 cell lines.

The cytotoxicity of compound **1** and FW9-10- F51-80 (compounds **4–6**) obtained from the winter sample; and F2 (compounds **1**, **2,** and **3**) and FS6-10-F25-29 (compounds **4–13**) obtained from summer sample were measured against three human cancer cell lines, MOLT-4, MCF-7 and HT-29 (Figure 5). Cisplatin was used as a positive control. The non-polar fraction F2 (compound **1**) obtained from the winter sample showed weak cytotoxic activity against HT-29 cell line with an IC_50_ value of 69.1 ± 12.8 μg/mL (means ± S.E.M.), while the non-polar fraction (F2: **1, 2** and **3**) obtained from the summer sample showed moderate cytotoxic activity against all three cell lines with IC_50_ values 33.9 ± 2.0, 50.9 ± 1.0 and 37.8 ± 4.9 μg/mL (means ± S.E.M.), respectively (Figure 5). After purification of fraction F2 and obtaining the three compounds **1, 2** and **3** from summer sample, we tested them against mentioned cell lines, and found that these pure compounds were not active (IC_50_ > 100 µM) against the cell lines. We combined these three compounds again with ratios of 1:2:3, respectively, and tested against three cell lines. This combination showed weak cytotoxic activity against MOLT-4 cell lines with IC_50_ value of 70.0 ± 8.9 μg/mL (means ± S.E.M.).

**Figure 6.**
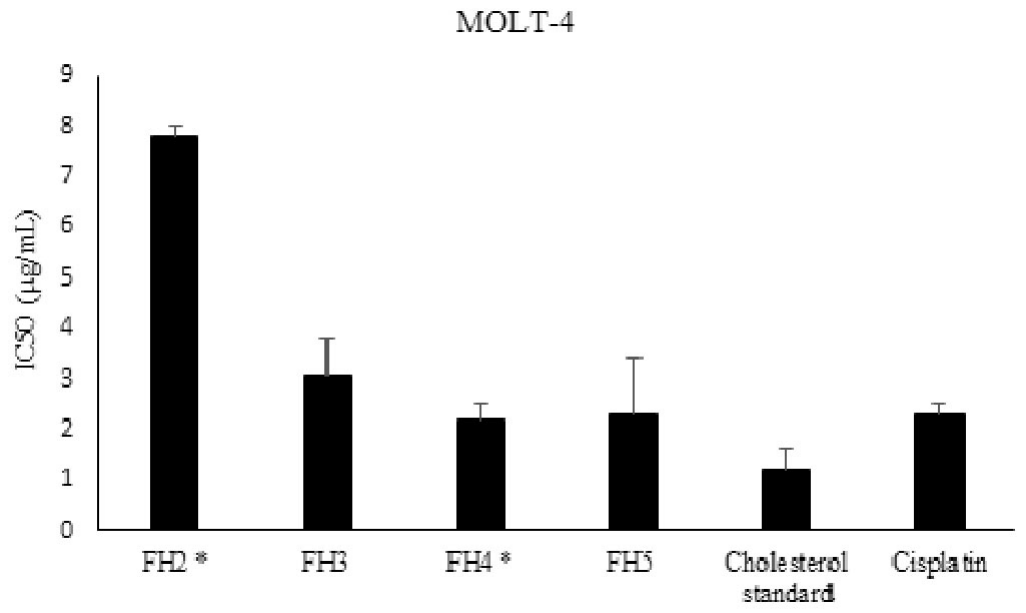
Cytotoxic activity of RP4-HPLC purified steroids isolated from *I. mutans* (summer sample) against human lymphoblastic leukaemia cell line. Values are presented as mean ± S.E.M. of 3-5 experiments. FH2: Compounds: **5**, **6**, **7** and **8**, FH3: compound **4**, FH4: compounds **4**, **9**, **10**, **11** and **12;** FH5: compound **13.** Based on statistical analysis, cytotoxic activity of FH2 has significant difference with other fractions (*p* < 0.05) while FH4 and FH5 were not significantly different compared to that of cisplatin as a positive control (*p* > 0.05).

Steroids from the winter sample FW9-10- F51-80 showed potent cytotoxic activity against all three tested cell lines with IC_50_ values of 13.0 ± 0.9, 11.1 ± 1.7 and 1.1 ± 0.4 μg/mL against the mentioned cell lines, respectively. Steroids from summer sample FS6-10- F25-29 just was tested against MOLT-4 cell lines and showed strong activity with an IC_50_ value of 1.1 ± 0.2 μg/mL. The cytotoxic activity of the steroids purified from the summer sample, FH2, FH3, FH4 and FH5 were evaluated against MOLT-4 cell line and showed that all steroids are potent cytotoxic compounds with IC_50_ values of 7.8 ± 0.2, 3.1 ± 0.7, 2.2 ± 0.3 and 2.3 ± 1.1 μg/mL, respectively (Figure 6).

## Discussion

Furodysin (1) was previously isolated from genus *Dysidea* and its structure was determined by single crystal x-ray crystallography (Kazlauskas et al. 1978). The full NMR spectroscopic data assignment of the *ent*-furodysin enantiomer, isolated from *Dysidea fragilis*, was performed by 1 D and 2 D NMR spectroscopy and found to be different in their ^1^H and ^13^C NMR δ values and optical rotation with those reported for compound **1** (Su et al. 2010). The structure of compound **2** isolated from summer sample was determined based on ^1^H and ^13^C NMR spectra and comparing them with those reported previously from a China Sea sponge, *Dysidea fragilis* (Su et al. 2010). The ^1^H and ^13^C NMR data of **1** and **2** are reported in the supplementary file of the paper. Compound **3** has similar pattern of fragmentation in the EI mass spectrum, but differences were found in the NMR spectral data with those reported for **1** and **2**. Compound **3** was previously synthesised in 1991 (Vaillancourt et al. 1991), but to the best of our knowledge it is the first report of its isolation from a natural source. Furanosesquiterpenes did not show a strong cytotoxic activity against tested cancer cell lines, however the greater cytotoxic activity of mixture of compounds **1–3** compared to those measured for the individual compounds suggested their synergetic effects in the mixture.

Steroids were the other dominant natural compounds extracted from the Persian Gulf sponge, *I. mutans*. Cholesterol (**4**) (36.8% area), cholesta-5,7-dien-3β-ol (**5**) (22.6% area) and ergosta-5, 7, 22-trien-3β-ol (ergosterol) (**6**) (10.7% area) were the major steroids from the winter sample. The main steroids detected in summer sample like winter sample consisted of **4** (46.8%), **5** (17.3%) and **6** (9.3%). The other steroids (**7–13**) were the minor steroids isolated from the summer sample of this sponge which were not detected in the winter sample. Ergosterol has been isolated from most calcareous sponges such as *Leucetta chagosensis* Dendy (Leucettidae), *Pericharax heteroraphis* Poléjaeff (Leucettidae), *Clathrina* sp. Grey (Clathrinidae), and *Grantiopsis* sp. Dendy (Lelapiidae) as their major constituent, and in many other species, cholesterol is reported as the minor sterol (Sica et al. 1987). While in our experiment, cholesterol was the major steroid.

Since Larak Island (26°51′12″N and 56°21′20″E) is located in a subtropical region, where the water temperature during winter (18 °C) is substantially lower than of the summer (29 °C), we can infer that the greater variety of isolated steroids and furanosesquiterpenes from the summer sample is related to water temperature, which of course is likely also correlated with great fouling and competition from other organisms.

In this research the steroids showed stronger cytotoxic activity than did the furanosesquiterpenes. Although steroids obtained from both seasons showed strong cytotoxic activity, it should be noted that the steroid fraction from the summer sample, which not only contains more diverse Δ^5^ and Δ^5,7^ steroids, but also comprises Δ^7^ sterols, showed stronger cytotoxic activity (IC_50_ values 1.1 ± 0.2 μg/mL) than the winter sample (IC_50_ values 13.0 ± 0.9 μg/mL) against MOLT-4 cell lines (*p* < 0.05). Various researches showed that environmental variations can affect the bioactivity of natural compounds. Turon et al. (1996) found clear annual pattern in toxicity of *Crambe crambe* Schmidt (Crambeidae) and showed the highest biological active in late summer and autumn, similar to the results reported here. Cholesterol with IC_50_ value 3.1 ± 0.7 μg/mL was one of the potent cytotoxic steroids which extracted from Persian Gulf sponge, *I mutans*. Kellner-Weibel et al. (1999), showed that free cholesterol generated by the hydrolysis of cytoplasmic cholesteryl esters in model macrophage foam cells, transported to the plasma membrane by acidic vesicles, and accumulation of this compound in the pool caused cell death by necrosis and apoptosis. γ-Sitosterol, IC_50_ value 2.3 ± 1.1 μg/mL was the other potent cytotoxic steroid which isolated from *I. mutans*. Sundarraj et al. (2012), investigated the inhibitory effect of *Acacia nilotica* Delile (Fabaceae) leaves extract and γ-sitosterol on cell proliferation, apoptosis and cell cycle arrest in breast and lung cancer cells, and showed that γ-sitosterol with induces G2/M cell cycle arrest and apoptosis through c-Myc suppression in MCF-7 and A549 cells was a potent anticancer agent.

Generally, we showed that steroids from *I. mutans* have strong cytotoxic activity on MOLT-4, MCF-7 and HT-29 cell lines with IC_50_ value <10 µg/mL. The steroids composition of the sponge, *I. mutans* is similar to those of its neighbouring sponge, collected from the same location, *A. sinoxea*. Both fractions and purified compounds have the same range of activity against the mentioned cell lines with IC_50_ values of 1.20–4.12 μg/mL (Jamebozorgi et al. 2019). Therefore, our present results confirmed the previous findings that suggested steroids from sponges as potent cytotoxic agents.

## Conclusions

The Persian Gulf sponge, *I. mutans*, is a rich source of valuable secondary metabolites. Steroids are one of the dominant groups of secondary metabolites found in *I. mutans* and they show strong cytotoxic activity against three human cancer cell lines. Therefore, they can be considered as candidates for further investigation as anticancer agents. Also, the study of the extracted metabolites of this sponge in the winter and summer season showed that both the diversity and cytotoxic activity of metabolite extracted in the summer was greater than winter season, However, additional sample collections and statistical analyses are needed to verify these differences in future research. Finally, compound **3** or its proper isomer may be considered as intermediates in the biosynthetic pathway of compounds **1** and **2**, respectively.
